# Case of Fracture and Suppuration around the Incisive Alveolar Portion of the Superior Maxillary Bone, and Reimplantation in the Same of a Lateral Incisor Eight Days after the Injury

**Published:** 1893-11

**Authors:** A. C. Hugenschmidt

**Affiliations:** Paris, France


### CASE OF FRACTURE AND SUPPURATION AROUND THE INCISIVE ALVEOLAR PORTION OF THE SUPERIOR MAXILLARY BONE, AND REIMPLANTATION IN THE SAME OF A LATERAL INCISOR EIGHT DAYS AFTER THE INJURY.[1]

BY A. C. HUGENSCHMIDT, M.D., D.D.S., PARIS, FRANCE.

In October, 1890, one Saturday afternoon a gentleman came to my consultation to present his son, a boy ten years old, with the following history:

On the preceding Monday, that is, five full days before our examination, this young boy, riding a bicyclette at a great speed, was suddenly thrown to the ground, face forward. The road was wet and very muddy. The apparent result of the injury at the time was a vertical wound of the upper lip, complete fracture of the left central incisor, almost total fracture of the right central, while the left lateral incisor had been thrown out of the mouth into the mud on the road. The father, searching later on on the spot of the accident, found the missing tooth, and after having wiped it placed it in his waistcoat pocket.

The lip was sutured by a surgeon of the neighborhood of the accident, who paid no attention to the buccal state of affairs, and the young fellow was left for several days until we saw him five days later.

As soon as he entered the office, what struck one immediately was a most penetrating and sickening odor, which had for origin the buccal cavity of the boy. On lifting the upper lip, still greatly increased in volume on account of the sustained injury, a large quantity of pus exuded from the socket of the left lateral incisor, which was absent, as well as from the margin of the gum surrounding the roots of the central incisors. These roots could be moved to such an extent that they appeared as if they would drop out of the alveoli.

Probing with an exploring needle in the alveolus of the left lateral incisor, hard, irregular fragments of bone were encountered, and we found in addition a fracture of the anterior alveolar wall. Two vertical fissures existed, one started immediately above the root of the right central incisor, while the other occupied the space between the alveolus of the left lateral incisor and canine of the

[1] Read before the Society of Stomatology of Paris.

same side. Moreover, to this fragment of bone was adherent, at its posterior surface, the root of the left central, the whole forming a unique mass quite detached from the body of the maxilla; from all these parts flowed extremely fetid pus. The first step in the treatment was to subject all these parts to a thorough disinfecting process with a solution of permanganate of potash, which was syringed through all the fissures which showed the least trace of pus, then boiled water was used to wash out the previous solution, and finally a series of injections of oxygenated water.

After having thoroughly disinfected all these parts, the young patient was sent away until the following Monday, recommending, however, to the father, that the boy should wash and bathe his mouth every half-hour during the day, alternately with a solution of bichloride of mercury 1 to 5000 and a saturated solution of boracic acid. I directed the father to apply during the night, around all the teeth, and especially in the region which had sustained the traumatism, the following preparation:

Boracic acid, ʒiss;

Vaseline, ℥i. M.

I must add that the father, before leaving me, had handed to me the lateral incisor, which he had kept in his waistcoat pocket ever since the accident. As I found, on examining the tooth, that no injury had been sustained by that organ, I determined to disinfect it as thoroughly as possible by opening the pulp chamber and by placing it in a solution of bichloride of mercury 1 to 1000.

On the Monday morning, eight days after the accident, the young man was brought back to me; the prescriptions had been carefully followed, for I tried my best to find a drop of pus in the region which two days before was absolutely saturated by it. The parts looking so well, I determined to insert the lateral incisor.

To be on the safe side, several injections of oxygenated water were made, followed by sterilized or boiled water; an injection of cocaine rendered the parts insensible. Finding the alveolus of the lateral obstructed by several spiculæ of bone, which had been detached from its anterior wall, these were removed with the engine-bur, until the cavity was free.

The lateral was then removed from the bichloride solution, the pulp cavity dried and filled with gutta-percha, and finally inserted in the alveolus. It was maintained in place by means of silk ligatures around the left canine and what remained of the right central incisor. This last, which two days before was bathed in pus, had already become consolidated. Moreover, the ligatures were placed in such a manner as to maintain not only the reimplanted tooth in its place, but also the part of the alveolus which had been detached and fractured at the time of the fall.

The only treatment recommended during the consolidation process was buccal lotions with boric-acid solutions in daytime, and boracic vaseline at night. Three weeks later the union was complete, the bone had united, and the tooth was firm in its place. The ligatures were removed. The tooth is in place to-day, two years after the accident.

The interesting part of this case is the extensive infection existing at the time the boy presented, and the insertion, only *two days later*, in that very time, of a dental organ, which did not produce the least inflammatory reaction or disturbance.

